# Determinants of delivery practices among Afar pastoralists of Ethiopia

**Published:** 2012-12-26

**Authors:** Medhanit Getachew Mekonnen, Kassahun Negash Yalew, Jemal Yesouf Umer, Muluken Melese

**Affiliations:** 1 Africa Medical and Research Foundation (AMREF), Ethiopia

**Keywords:** Traditional birth attendant, antenatal care, educated mother

## Abstract

**Introduction:**

In a previous qualitative study in Afar, it was reported that most mothers deliver at home, assisted by Traditional Birth Attendants (TBAs). However, determinant factors of delivery practices in this region remain scarcely explored. The objective of this study was to elucidate these factors among the Afar community.

**Methods:**

This cross sectional study was conducted in April 2011 in zone 3 of Afar regional state, Ethiopia. Quantitative data were collected from 478 women who had given birth during the preceding one year.

**Results:**

Out of 478 interviewed mothers in urban/rural areas, 398 (83.3%) gave birth to the youngest child while at home; 370 (92.5%) were assisted by TBAs. Only 3.2% of them were assisted by Health Extension Workers/nurses in health posts or at home during delivery. We found an association between health facility delivery and ante-natal care (ANC) attendance (p<0.001), educational status (p<0.001) and occupation of the husband/wife (mother) and gravidity (p=0.003); but there was no association with the number of wives the husband had (p=0.566). In the adjusted model, ANC attendance and education status of mother were significantly associated with health facility delivery (p=0.036; p<0.001).

**Conclusion:**

Most deliveries in the study community took place at home. Educated mothers and ANC attending mothers have high tendency for health facility delivery. Programmes need to strengthen the capacities of mothers to attend ANC services, as well as build the capacity of Health Extension Workers (HEWs) and nurses working in health posts, in order to win the confidence of the community.

## Background

More than 90% of maternal deaths in the world occur in Sub-Saharan Africa (SSA) and Asia, whilst women in northern Europe have a 1 in 4,000 likelihood of dying from pregnancy related causes. For those in Africa, the chance is 1 in 16 [1]. Most of these deaths occur because of distant health facilities; lack of transport; high cost of service for mothers and inadequate skilled attendants; poorly motivated staff; inadequate equipment and supplies; and weak referral systems [2].

In low and middle income countries, most deliveries take place at home without the assistance of trained attendants [3–5]. Although the annual maternal deaths decreased from 526,300 in 1980 to 342,900 in 2008, only 23 countries have met the Millennium Development Goal (MDG 5) [6]. The MDG 5 is to reduce maternal mortality ratio by 75 per cent by 2015 [7]. The World Health Organization indicated that each year about 3.7 million children die within the first 28 days and close to 9.7 million children die before their fifth birthday [8]. UNICEF stated that progress towards reaching MDG 4, the goal of reducing the under-five mortality rate by two thirds by 2015, is “insufficient” in the Middle East and North Africa, SSA and South Asia [9]. The burden of maternal mortality remains greatest in SSA [10]. Studies have shown that around 20 - 30% of neonatal deaths can be averted if deliveries are attended by skilled birth attendants [11].

Home deliveries are common in developing countries. Studies in some countries reported that home deliveries range from 16% in Mumbai (India), 22% in Senegal, 65% in Tanzania to 87.7% in Bangladesh [12–15]. The main reasons given by mothers for home deliveries is distance to health facilities [13, 14, 16, 17]; cost for medical care; delivery complications; low education level and absence of ANC [18, 19]. This study was designed to collect data on the rate of home delivery and the determining factors for institutional delivery, among women in rural and urban areas of zone 3 of Afar region.

## Methods

### Study area

This study was conducted in Zone 3 of the Afar regional state of Ethiopia. This Zone is divided into six districts namely Awash, Amibara, Birumodytu, Gewane, Dulessa and Argoba special districts. The total population of Afar regional state is 1,418,000 and the study zone population is nearly 200,000 [20]. Afar regional state is located in the eastern part of Ethiopia and lies in the East African Great Rift Valley. In the west it is bordered by Tigray, Amhara and Oromia regional states; in the south by Somali regional state; in the east by Djibouti and in the north by Eritrea. It is a semi-arid region and more than 90% of the population is pastoralist.

### Study design and sample size

A cross-sectional design was employed for interviewing women, between the ages of 15 - 49 (reproductive age groups) who delivered within the last one year, on determinants of delivery practices.

The sample size was calculated using Epi-Info version 3.3 for Windows for single proportion formula. Calculation was based on the assumption of Type I Error of 5%, reported delivery in health facilities of 12.9% (Afar Regional Health Bureau 2006/2007 report), and a designing effect of 3 with 5% non response rate. The total sample size was 502 women between the ages of 15 - 49 years. A three stage sampling strategy, that is, districts to kebele then household was used to reach the target population.

Estimated household size in each district was obtained from the National Population and Housing census report. The number of women to be interviewed (502) was allocated equally to 4 districts; these districts were randomly selected out of 6 districts of the Zone. Then, 12 kebeles (smallest unit of public structure and possibly less than a village) were randomly selected in each district and the allocated households for the district were equally distributed amongst the kebeles (which amounted to about 11 mothers per kebele). In each selected kebele, the centre of the kebele was identified as the starting point and a bottle was spun to determine the direction to be followed. The survey then started from the nearest house to the direction of the bottle top, and continued in the determined direction. Households were surveyed sequentially until the desired number was obtained. All women in the age category of 15 - 49 years, from the sampled households, who gave birth during the last one year, were interviewed using the survey questionnaire.

Data collectors who had previous experience of data collection were recruited and trained for two days on how to use the questionnaire. The questionnaire was initially developed in English and translated to Amharic for data collection and translated back to English. It was tested and finally all findings from the pre-test were incorporated into the final form of the questionnaire.

To estimate the rural households’ asset levels, a wealth ranking was conducted, based on the following five categories, namely poorer (cows/oxen 0, camels 0, sheep/goats less than 5); poor (cows/oxen less than five, camels 0, sheep/goats between 5 - 9); middle (cows/oxen 5 - 9, camels 1 - 5, sheep/goats 10 - 19); rich (cows/oxen 10 - 29, camels 5 - 9, sheep/goats 20 - 49) and richer (cows/oxen 30 and above, camels 10 and above, sheep/goats 50 and above). During the data collection, the number of animals for each category was collected from the sampled households.

### Data Quality Control and Analysis

During data collection, supervisors watched over the data collectors on site and every evening checked the data for accuracy, consistency and completeness. Supervisors also rechecked five percent of the samples in order to cross check the collected data. The data were entered and analyzed in SPSS software (version 16). The association between the socio-demographic and reproductive variables, ANC attendance and delivery place were analyzed using Pearson's Chi-square test. Logistic regression analysis was also used to measure unadjusted and adjusted odds ratios to identify the predictors of health facility delivery.

The asset level for the rural dwellers was calculated, taking the average market value of each category of the animal in Birr to get the total asset value of the household. However, during analysis it was found that the middle and rich categories were few; therefore for convenience of the analysis, the data was merged into rich and poor only. The richer, and the rich categories were merged into rich and the others were merged into poor.

### Ethical Clearance

A written permit was obtained from Afar Regional Health Bureau and verbal consent from interviewed mothers.

## Results

### Socio-demographic characteristics

Out of the sampled 502 mothers who delivered during the last one year, 478 (95%) were interviewed. Out of all those interviewed, 347 (72.7%) belonged to the Afar ethnic group while Amhara accounted for 47 (9.8%); the rest, 83 (17.8%), belonged to various other groups. The majority of women, 468 (97.3%), were married and only 1.7% were single. The socio-demographic characteristics are presented in Table 1.

**Table 1:** Characteristics of respondents for home and institutional delivery

Characteristics	Home delivery	Institutional delivery	Pearson Chi-square	p-value
**Women's Age**			14.29	0.003
15 – 18	11	7		
19 – 24	114	30		
25 – 29	110	23		
≥30	163	18		
**Gravidity/number of total pregnancies**			18.39	0.031
0	0	0		
1	74	22		
2	78	22		
3	77	18		
4	53	10		
5	45	4		
≥6	62	2		
**Parity/number of living children**			15.72	0.07
0	0	0		
1	83	24		
2	80	20		
3	79	19		
4	51	9		
5	45	4		
≥6	48	1		
**Number of wives a husband has**			2.03	0.566
1	349	71		
2	40	6		
3	7	0		
4	1	0		
**ANC attendants**			26.3	<0.001
Yes	233	70		
No	160	8		

### Delivery place

Out of the 478 mothers interviewed about the place of their last delivery, 398 (83.3%) had delivered at home and the remaining 16.7%) had delivered in health facilities. Among women who delivered at home 370 (92.5%) of them were assisted by TBAs and about 3.6% were assisted by Health Extension Workers/nurse; a further 3.2% were assisted by a neighbor. Out of all the reasons given as to why they preferred home deliveries, 205 (51.5%) of them said that it was because labour progressed fast and there was no time to reach a health facility. Cultural ceremonies surrounding home deliveries and lack of confidence in health facilities were not reasons for opting for home deliveries (Table 2).

**Table 2:** Socioeconomic characteristics of the respondents

Characteristics	Home delivery	Institutional delivery	Pearson Chi-square	p-value
**Educational Status of mother**			41.67	<0.001
Cannot read and write	324	39		
Read and write only	10	2		
Primary school 1 – 8	43	15		
Secondary school 9 – 10	16	12		
Higher education/university/college 10+	4	6		
**Educational Status of husband**			53.9	<0.001
Cannot read and write	285	24		
Read and write only	27	8		
Primary school 1 – 8	32	18		
Secondary school 9 – 10	33	14		
Higher education/university/college 10+	15	12		
**Occupation of mother**			18	0.003
House wife	352	58		
Government/private employee	22	13		
Merchant	5	2		
Pastoralist	6	0		
Semi-pastoralist	2	2		
Other	11	3		
**Occupation of husband**			58.4	<0.001
Farmer	25	3		
Government/private employee	116	4		
Merchant	45	5		
Pastoralist	10	1		
Semi-pastoralist	101	53		
Daily labourer	64	5		
Job less	19	3		
Other	14	4		
**Monthly Income (Birr)**			24.25	<0.001
<500	100	16		
500-1000	44	25		
1001-2000	4	7		
>2000	0	1		

As shown in Table 2, there was an association between home delivery and age (p=0.003), educational status and occupation of the husband/wife (mother); p<0.001, and gravidity and parity (p<0.031), but there was no association with the number of wives the husband had (p>0.566). Out of 197 urban mothers interviewed, 49 (24.8%) of them had delivered in health facilities. Moreover, a higher economic status increased the likelihood of health facility delivery; p<0.001 (Table 2). There was no association between economic status and health facility delivery in the rural set up when the rural and urban income level data were analyzed separately viz a viz health facility delivery. Additionally, there was also an association between mothers’ educational status and health facility delivery (p<0.001).

The choice of TBAs is mostly related to the emergency nature of deliveries. The reasons for mothers’ choice of health facility for their delivery were: fear of bad outcome for themselves and the fetus, 42 (45.7%); referral by TBAs, 19 (20.7%); referral by HEWs, 9 (9.8%) and previous risk of adverse outcome during home delivery 16 (17. 4%).

### Antenatal Care (ANC) Practices

Of the 478 women who were asked about their ANC follow up for their last pregnancy, 305 (63.8%) had attended ANC at least once during their pregnancy. For those who did not attend ANC, 48 (50%), as described in Table 3, the main reason for not doing so was mainly related to distance of health facilities and a few, 12 (13%), reported that there was no advantage of ANC.

**Table 3:** Reasons given for home delivery

Reason	Frequency	%
Labour too quick to reach health institution	205	51.51
Health facilities are too far	61	15.33
Home delivery is more comfortable	52	13.1
No transport	26	6.53
Health workers are not friendly	20	5.02
Home deliveries allow family and friends to attend the delivery	12	3
Cost of medical services	6	1.51
Health facilities are not clean	2	0.5
Missing data	14	3.5
**Total**	**398**	**100.0**

Most of the interviewed mothers, 425 (88.9%), reported that every pregnancy has a risk for the mother and the fetus. As shown in Table 4, there was a significant association between attending ANC and knowing the advantages of ANC (p<0.001), but there was no significant association between knowing pregnancy/delivery danger signs and ANC attendance (p>0.05), except for difficulty of breathing in pregnancy (p=0.03).

**Table 4:** The relationship between knowing risk factors of pregnancy/delivery and attending ante-natal care

Characteristics	Number	% women who attended ANC	Pearson Chi- Square	p-Value
**Advantages of ANC**				
Assess maternal health	374	75.1	90.9	<0.001
Assess fetal health	272	77.9	49.8	<0.001
Assess fetal position	183	84.2	50.28	<0.001
Assess possible delivery complication	111	86.5	30.55	<0.001
No advantage	9	22.2	7.18	0.007
I don't know any advantage	63	6.3	106.9	<0.001
Other	7	57.1	0.17	0.68
**Danger signs of pregnancy/delivery**				
Bleeding during pregnancy	215	68.4	2.36	0.124
Fever	147	68.7	1.52	0.218
Convulsion	47	72.3	1.34	0.247
Difficulty in breathing	37	81.1	4.7	0.03
High blood pressure	18	72.2	0.47	0.495
Edema of hands and face	104	68.3	0.75	0.386
Decreased/absent fetal movement	15	60.0	0.15	0.7
Labour more than 12 hours	56	64.3	0.00	0.966
I don't know	50	56.0	1.85	0.174
Other	49	65.3	0.01	0.923

Concerning the knowledge and skills of HEWs in conducting ANC, 269 (56.3%) of the mothers said that HEWs were superior to TBAs in terms of knowledge and skills; 96 (20.1%) said that TBAs were superior; 64 (13.4%) were not able to compare; 22 (4.6%) said that HEWs and TBAs were not different while 19 (4%) said HEWs could not conduct ANC.

### Predictors of using health facility delivery

A logistic regression model was applied for variables which showed significant association. These variables were gravidity, age of mothers, ANC attendance, occupation and educational status of husband/wife. Education status of wife and ANC attendance among mothers showed significant association from all variables with p=0.036 and p<0.001 levels of significance respectively.

## Discussion

Most mothers in the study community (83.3%) had delivered at home; less than 25% had delivered in health facilities. The main reason given for home deliveries was that the emergency nature of delivery did not allow the mothers to be transported to health facilities. Most mothers live in the rural areas far from health facilities, and there was no transport system. Studies in other countries have also reported similar findings, whereby distance plays the main part in determining health facility delivery [10, 13, 16, 17]. In this study, home ceremonies during delivery were not found to be determinant factors for home deliveries in this study. Traditional birth attendants are still the main attendants whilst there are HEWs and nurses in some of the study kebeles. The findings show that most mothers believe that HEWs are superior to TBAs in terms of skill; however, a significant proportion also prefers TBAs and some are even doubtful about the skills of HEWs. In a previous qualitative study in the same area, participants reported that they had no confidence in HEWs. The reason being that HEWs’ training is short; they are young and have not proven themselves like TBAs [21]. The difference in the reasons given between the two studies may be due to social desirability bias in the quantitative study. Considering that the data collectors came from the government, labeling HEWs as “lower than TBAs” may have been seen to be opposing the government initiative. This is an area which needs intervention in order for the community to develop confidence in HEWs assigned to health posts. One possibility is to bring the labouring mother to the health posts along with the TBA so that both the TBA and the HEWs can assist delivery together. This would give a better chance of a clean delivery at a health post and would enable a referral to be organized if the mother needs one.

In this study, we found an association between ANC attendance and delivering in health facilities. In the urban areas, delivery is significantly associated with the income of the household, whilst it is not a factor for the rural. This is probably because in the rural settings, whether the mother has money or not, there is no transport and no time to take a labouring mother to health facilities. Again, as reported in our previous study, it was only when the mother failed to deliver at home that the community took her to a health facility (authors’ AMREF unpublished data). This does not require a lot of money, as it is a communal responsibility. In health facilities, delivery is free unless the mother needs to go to a referral hospital wherein the family would incur transport costs.

Although there is an association between status of education and health facility delivery, ANC and knowing the advantage of ANC, there was no association between delivery in health facilities, and knowing the danger signs in pregnancy/ delivery [18, 19]. This could have been due to the distance of health facilities for most mothers as there was no chance even if they wanted to go because there was no transport system to take the mother to health facilities [12–14]. Thus, there is need to design an affordable and appropriate transport system for the area. Rickshaw ambulances can work for Afar as most of the area is flat. Rickshaws are cheap and their maintenance cost is low. The other option is to organize a camel-pulled cart. Ambulances that can be stationed in health centres to transport mothers from health centres to hospitals are crucial. Hospitals are more than 200 kilometres from the study areas.

Based on the findings of this study, several recommendations can be made. The first recommendation is to teach mothers on the importance of ANC and institutional delivery. Along with this, developing the competence of HEWs and equipping health posts are important steps in bringing skilled birth attendance nearer to the community. TBAs are still much preferred by the community for good reasons. There is no way for HEWs to get the community's confidence over TBAs in the short term. We need to understand and communicate the concerns of the community. A middle ground strategy of encouraging TBAs to attend all deliveries in health posts, together with HEWs, should be designed. This way, clean delivery can be offered to the mothers. If the community ambulance system, as mentioned above, is attached to health posts, mothers with difficult labours can be referred in time to health centres. In the towns, deliveries still take place at home and there is need to educate the mothers. Ambulance service for health centres will facilitate the transportation of mothers to hospitals.

## Conclusion

This study reports the rate of home delivery and the determinant factors of institutional delivery in Afar region. Majority of deliveries in the study area took place at home mainly assisted by TBAs. Knowledge of danger signs in pregnancy was insignificant for institutional delivery. However educated mothers and mothers who attended ANC had a higher tendency for health facility delivery.

Therefore, there is need to educate mothers on the importance of ANC and institutional delivery. Additionally, developing the competence of HEWs and equipping health posts are important steps in bringing skilled birth attendance nearer to the community. As TBAs are still much preferred by the community for good reasons, it may be difficult for HEWs to get the community's confidence over TBAs in the short term. Importantly, the community's concerns should be communicated to local health system's stakeholders. A middle ground strategy of encouraging TBAs to attend all deliveries in health posts, together with HEWs, should be considered. This is likely to result in increased confidence among community members on health facility delivery; and in the long-term result in better maternal outcomes.
